# Hardware-preserving management of early soft-tissue breakdown over stable osteosynthesis: a retrospective single-centre experience

**DOI:** 10.1007/s00590-026-04800-3

**Published:** 2026-06-08

**Authors:** Ferruccio Paganini, Sara Matarazzo, Valentina Ceretti, Elisa Bascialla, Beatrice Corsini, Martina Corno, Silvia Cozzi, Luigi Valdatta

**Affiliations:** University of Insubria, Varese, Italy

**Keywords:** Hardware exposure, Hardware preservation, Soft tissue breakdown, Osteosynthesis, Orthoplastic, Negative pressure wound therapy, Flap reconstruction, Wound dehiscence, Retrospective study, Limb fractures

## Abstract

This study evaluates whether a hardware-preservation strategy—restoration of soft tissue coverage over exposed but mechanically stable osteosynthesis combined with systemic antibiotic therapy aimed at limiting progression from contamination to established infection—can represent a viable alternative to hardware removal in selected patients with orthopaedic hardware exposure without overt clinical signs of infection and with negative superficial wound swabs. A retrospective analysis at Circolo and Macchi Foundation Hospital (January 2018–January 2023) examined all cases of soft tissue breakdown over orthopaedic hardware in limb fractures treated with plates. Inclusion criteria required absence of overt clinical signs of infection, negative superficial wound swabs, stable fixation, and no prior plastic surgery at the same site. Treatment was classified as outpatient management (OM: dressings or negative pressure wound therapy) or surgical management (SM: soft tissue reconstruction without hardware revision). Associations with failure were explored using Fisher’s exact test and the Mann–Whitney U test. Twenty-seven patients (median age: 74 years) were included, with 93% of exposures involving the lower limb. Hardware-preservation success was achieved in 24 patients (89%); final functional recovery, including cases requiring subsequent revision, was observed in 26 (97%). All three failures occurred at the malleolar site, involved exposures exceeding 1 cm, and two of three presented after one week. Time to treatment beyond one week showed the most pronounced trend (25.0% vs 5.3%; *p* = 0.201; OR 6.0), followed by malleolar location (18.8% vs 0%; *p* = 0.248); neither reached statistical significance. These preliminary findings suggest that hardware preservation may be feasible in selected patients with stable fixation and no overt infection. Failures clustered in malleolar exposures larger than 1 cm, particularly when referral occurred after one week; however, these observations remain hypothesis-generating and require validation in larger, anatomically focused studies.

## Background

Despite the increasing number of osteosynthesis procedures performed worldwide, the management of soft tissue breakdown over orthopaedic hardware remains controversial [1, 2]. In particular, there is a lack of high-quality, evidence-based protocols for managing hardware exposure in the absence of clinically manifest infection. Most of the current literature advocates for hardware removal as the standard of care, based on the widespread assumption that exposure equates to infection and that any exposed implant is, by definition, compromised beyond salvage. However, this approach may not always be justified.

While it is reasonable to assume that any exposed implant is at least contaminated, contamination does not necessarily equate to established infection. Clinical experience suggests that, in selected cases, when fixation remains mechanically stable and no overt clinical, laboratory, or radiographic signs of infection are present, bone healing may proceed successfully without removing or replacing the hardware, provided that soft tissue coverage is restored and systemic antibiotic therapy is maintained to limit progression from contamination to established infection. The literature on this subject remains sparse, with only one systematic review addressing reconstructive strategies aimed at hardware preservation [3]. As a result, there are no well-established criteria to guide the selection of patients who may benefit from a hardware-preservation strategy.

### Study objectives

This retrospective, preliminary observational study aims to evaluate the feasibility and outcomes of a hardware-preservation management strategy—defined as the restoration of soft tissue coverage over exposed but mechanically stable osteosynthesis, combined with systemic antibiotic therapy—in patients without overt clinical signs of infection and with negative superficial wound swabs.

Traditionally, the detection of hardware exposure prompts aggressive treatment, including removal of the implant, bone and soft tissue debridement, and subsequent reconstruction. These procedures often result in substantial soft tissue defects requiring flap coverage [4, 5]. Based on the prevailing notion that any exposure mandates hardware replacement, surgeons frequently proceed with extensive surgical interventions—even in the absence of clear clinical, laboratory, or radiographic evidence of infection.

Nonetheless, this protocol is not universally adopted. At our institution, we observed a subset of patients with localized hardware exposure in the early postoperative period, with stable fixation and no signs of established infection, who responded favourably to a hardware-preservation approach—targeting the soft tissue defect while maintaining the existing osteosynthesis under systemic antibiotic therapy. To date, however, the literature offers minimal guidance on which patients are most likely to benefit from such an approach.

The primary objective of this study is therefore to describe our institutional experience with hardware preservation in the setting of early postoperative soft tissue breakdown, and to assess whether this strategy can represent a viable alternative to hardware removal in selected cases. The secondary objective is to identify clinical and procedural factors—such as time to intervention, exposure size, and anatomical site—that may be associated with the success or failure of this treatment. Given the small sample size, this work is intended as a hypothesis-generating preliminary study rather than a definitive validation of a treatment algorithm.

### Terminology clarification

The management of soft tissue breakdown over stable osteosynthesis can be pursued through interventions of varying complexity. To allow for stratification of treatment modalities and evaluation of their respective outcomes, we classified the hardware-preservation strategies employed in this series into two categories based on the treatment setting and level of intervention:

*Outpatient Management (OM)*: a non-operative strategy performed in an outpatient setting, which includes advanced wound care techniques such as serial dressings, application of collagen-based or silver-impregnated hydrofibers, and/or the use of negative pressure wound therapy (NPWT). This modality aims to promote granulation and secondary coverage of the exposed implant without surgical intervention.

*Surgical Management (SM)*: an operative strategy requiring hospital admission and access to the operating theatre, including surgical wound irrigation, debridement, and direct closure or coverage using reconstructive techniques—such as local, locoregional, or microsurgical flaps—while preserving the original osteosynthesis. Although SM involves surgical intervention on the soft tissues, it is classified within the hardware-preservation spectrum because the defining feature of this approach is the maintenance of the existing bone fixation.

## Patients and methods

A retrospective analysis was conducted on all patients treated for soft tissue breakdown over orthopaedic hardware by the Departments of Orthopaedics-Traumatology, Hand Surgery, and Plastic and Reconstructive Surgery at Circolo and Macchi Foundation Hospital. Demographic, clinical, and procedural data were collected from all surgical and outpatient reports issued between January 2018 and January 2023 (Table 1).

**Table 1:** Inclusion and exclusion criteria

Selection criteria	
Time period	From January 2018 to January 2023
Exclusion criteria	Overt infection or positive superficial wound swabs
	Sub-optimally managed Gustilo type 1 or 2
	Gustilo type 3
	Time to plastic surgery evaluation longer than 2 weeks
	Paediatric patients
	Critically ill patients
	Hardware loosening
	Already achieved bone healing

**Fig. 1:**
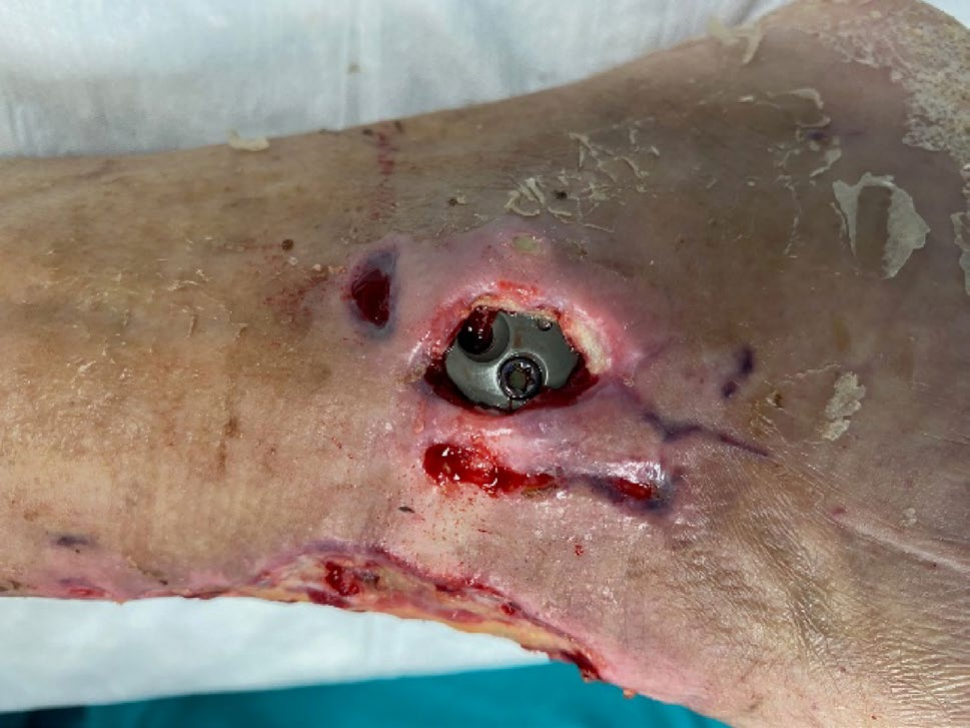
Exposure of approximately 1 cm over the medial malleolus plate on postoperative day 4. The wound shows dehiscence with visible hardware in the absence of clinical signs of infection

**Fig. 2:**
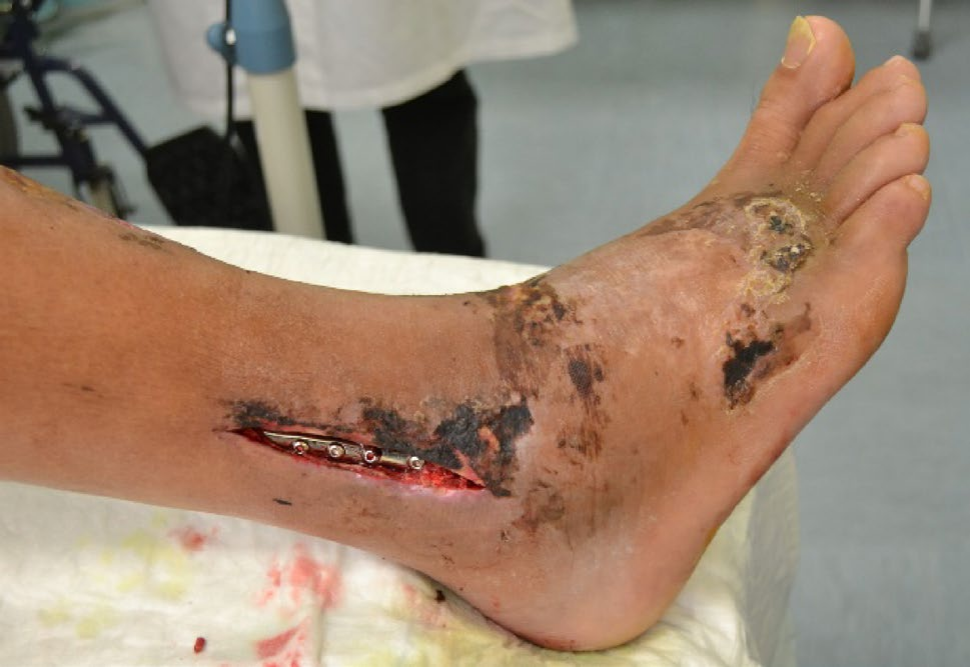
Exposure of lateral malleolar/distal lateral leg hardware on postoperative day 13, with dimensions exceeding 3 cm. Given the significant size of the exposure and the timing of presentation, hardware preservation was deemed unsuitable and the patient was referred for hardware removal and revision

The standard institutional pathway for patients presenting with hardware exposure can be summarized as follows. Following admission to the Orthopaedics Unit and initial management of bone synthesis for acute trauma, detection of skin necrosis or wound dehiscence over the implant (Figs. 1 and 2) prompted a comprehensive multidisciplinary evaluation involving both the orthopaedic and plastic surgery teams. This evaluation determined whether the patient should undergo hardware removal and revision of bone synthesis, or whether the exposure could be managed through a hardware-preservation strategy, either via outpatient management (OM) or surgical management (SM).

Contraindications to hardware preservation were established during the multidisciplinary discussion. These included the trauma’s aetiology (Gustilo type 3 open fractures or sub-optimally managed Gustilo type 1 and 2 open fractures), time elapsed from exposure detection to plastic surgery evaluation longer than 2 weeks, and local presentation of suspected or overt infection [6]. Additionally, patients with compromised systemic conditions, paediatric patients, cases of hardware loosening, or those with already consolidated bone were excluded from hardware preservation and referred directly to the Orthopaedics Unit for synthesis renewal or removal.

Patients were eligible for hardware preservation if they met all of the following criteria: hardware exposure affecting a plate or screw following open reduction and internal fixation (ORIF) for limb fractures; no previous plastic surgery procedures at the same anatomical site; absence of overt clinical signs of infection (erythema, purulent discharge, systemic fever); negative superficial microbiological wound swabs; fracture originally classified as Gustilo-Anderson type 1 or 2, managed according to established standards [7, 8]; referral to the Plastic Surgery Unit within 14 days of exposure detection; mechanically stable fixation with no radiological evidence of fracture consolidation at the time of evaluation. It should be noted that superficial wound swabs have a recognised limitation in sensitivity for detecting deep periprosthetic contamination; however, they were used in this series as a practical screening tool consistent with routine clinical practice, and their results were interpreted in conjunction with clinical assessment rather than as a standalone diagnostic criterion.

Within the hardware-preservation framework, two management lines were pursued: outpatient management (OM) and surgical management (SM). OM involved serial dressings (curettage of wound margin edges, washings with antiseptic solution, application of paraffin gauze and/or dressings with collagen or silver-based hydrofibers) or application of negative pressure wound therapy. SM required hospital admission and access to the operating theatre for wound irrigation, debridement and direct closure or flap reconstruction (local, locoregional, or microsurgical) without hardware renewal.

None of the patients in this series required offloading or pressure-relief devices, as no exposures occurred in weight-bearing or pressure-prone areas. Internal fixation in this cohort did not involve regions at high risk of pressure-related complications.

Local tissue perfusion was assessed clinically through the presence of active bleeding upon mechanical stimulation. When bleeding was absent or minimal, surgical curettage or debridement was performed until healthy, bleeding tissue was encountered, ensuring adequate vascularization for coverage procedures.

All patients received systemic antibiotic therapy, initiated at the time of exposure detection and aimed at limiting progression from contamination to established infection—consistent with the assumption that any exposed implant should be considered at least contaminated. The standard regimen consisted of amoxicillin and clavulanic acid 1 g three times daily or, in case of documented allergy, ciprofloxacin 500 mg twice daily, maintained for 21 days unless complications arose.

The evaluated variables were categorized into three groups (Table 2): demographic data and risk factors; fracture characteristics, treatment, and exposure features; type of hardware-preservation strategy performed, its outcome and follow-up.

**Table 2:** Evaluated variables

Demographic and risk factors	Trauma and subsequent exposure	Hardware-preservation strategy and outcome
Gender (Male, Female)	Fracture exposure (Closed, Open)	Management (OM, SM)
Age	Fracture location (Malleolus, Fibula, Patella, Elbow, Metatarsal)	Complications (Failure, revision)
Smoking	Timing of Plastic Surgery Unit intervention (< 1 week, > 1 week)	Recovery (Complete, Partial, None)
Diabetes	Exposure dimension (< 1 cm, 1-3 cm, > 3 cm)	Follow-up (> 2 years)
PVD		

Recovery was defined as a complete or partial return to daily activities, free of pain, with fracture healing time comparable to that of unexposed cases. Failure of hardware preservation was defined by non-healing of the dehiscence, overt infection, or delayed bone consolidation observed on subsequent imaging examinations. In such cases, treatment consisted of bone and soft tissue debridement, renewal of orthopaedic synthesis and soft tissue reconstruction.

Data were collected through the consultation of digital outpatient records and digitized operative reports. It should be acknowledged that the COVID-19 pandemic, which overlapped with the study period (2018–2023), may have resulted in incomplete case capture, as disruptions in outpatient follow-up and documentation could have led to underestimation of both the sample size and the true incidence of hardware exposure managed through this pathway.

Statistical analysis was primarily descriptive, given the limited sample size (n = 27) and the low number of outcome events (3 failures). Categorical variables were reported as absolute frequencies and percentages, and continuous variables as mean, median, and range. Associations between categorical variables and treatment failure were explored using Fisher’s exact test, appropriate for small samples with expected cell counts below 5. The comparison of age between the failure and success groups was performed using the Mann–Whitney U test. All p-values were two-sided, and a threshold of 0.05 was used for statistical significance. Given the exploratory nature of this analysis, no adjustment for multiple comparisons was applied, and the results of inferential tests are reported as hypothesis-generating rather than confirmatory.

## Results

A total of 27 patients treated from 2018 to 2023 were included, comprising 17 males and 10 females, with a median age of 74 years (mean 71, range 54–87 years).

Among these patients, 23 had at least one identified risk factor, including smoking habit (n = 15, 56%), diabetes (n = 8, 30%), or peripheral vascular disease (n = 14, 52%), with 10 patients presenting with two risk factors, and 2 patients having three risk factors simultaneously. Four patients had no identified risk factors (Table 3).

**Table 3:** Demographic and risk factors

	Total n (%)	Failure n/N (%)	*p*
Gender			1.000
Male	17 (63)	2/17 (11.8)	
Female	10 (37)	1/10 (10.0)	
Age			0.313
Median (range)	74 (54–87)		
Mean	71		
Failures: median 66 (59–68)			
Smoking			0.569
Active	15 (56)	1/15 (6.7)	
Non-smoker	12 (44)	2/12 (16.7)	
Diabetes			0.532
Yes	8 (30)	0/8 (0)	
No	19 (70)	3/19 (15.8)	
PVD			0.596
Yes	14 (52)	1/14 (7.1)	
No	13 (48)	2/13 (15.4)	

The vast majority of recorded exposures (25/27, 93%) occurred in the lower limb, with a notable concentration in the distal leg, between the distal fibula and the malleolar region. The malleolus was the single most common site, accounting for 16 cases (59%), followed by the fibula (6, 22%), patella (2, 7%), elbow (2, 7%), and metatarsal (1, 4%). Hardware exposures on originally open fractures were observed in only two cases (one Gustilo type 1 and one Gustilo type 2), both of which achieved healing with the hardware-preservation strategy, one through outpatient management (OM) and the other through surgical management (SM) (Table 4).

**Table 4:** Fracture characteristics and exposure

	Total n (%)	Failure n/N (%)	–
Fracture exposure			
Closed	25 (93)	3/25 (12.0)	–
Open	2 (7)	0/2 (0)	
Gustilo 1	1 (50)		
Gustilo 2	1 (50)		
Gustilo 3 A, B, C	0 (0)		
Fracture location			0.248
Malleolus	16 (59)	3/16 (18.8)	
Fibula	6 (22)	0/6 (0)	
Patella	2 (7)	0/2 (0)	
Elbow	2 (7)	0/2 (0)	
Metatarsal	1 (4)	0/1 (0)	
Timing of Plastic Surgery Unit intervention			0.201
< 1 week	19 (70)	1/19 (5.3)	
> 1 week	8 (30)	2/8 (25.0)	
Exposure dimension			1.000
< 1 cm	4 (15)	0/4 (0)	
1–3 cm	20 (74)	2/20 (10.0)	
> 3 cm	3 (11)	1/3 (33.3)	

Nineteen patients (70%) were managed with OM (4 with serial dressings and 15 with NPWT) and 8 (30%) with SM (7 local flaps and 1 free flap). Nineteen patients (70%) were referred to the Plastic Surgery Unit within one week of exposure detection, while 8 (30%) were referred between one and two weeks. The majority of exposures (20/27, 74%) measured between 1 and 3 cm, with 4 (15%) smaller than 1 cm and 3 (11%) larger than 3 cm.

Out of the 27 patients, 24 (89%) obtained coverage of the exposure with the proposed hardware-preservation approach. Three failures were observed, all occurring at the malleolar site, all involving exposures larger than 1 cm, and two of three presenting after one week from exposure detection. The first was a 59-year-old male with peripheral vasculopathy, malleolar exposure of 1–3 cm on a closed fracture, referred within one week, and treated with NPWT. The second was a 66-year-old female smoker, with malleolar exposure exceeding 3 cm on a closed fracture, referred after one week, and treated with SM and local flap reconstruction. The third was a 68-year-old male with no identified risk factors, malleolar exposure of 1–3 cm on a closed fracture, referred after one week, and treated with SM and local flap.

All three patients underwent orthopaedic surgical intervention for the removal of exposed hardware and the renewal of synthesis. Of these, two ultimately achieved bone and soft tissue healing following revision; one patient did not heal and underwent arthrodesis. Hardware-preservation success was therefore achieved in 24 of 27 cases (89%). Final functional recovery, including patients who required subsequent hardware revision, was observed in 26 of 27 cases (97%) (Table 5).

**Table 5:** Hardware-preservation strategy and outcome

	Total n (%)	Failure n/N (%)	p
Management			0.201
Outpatient management (OM)	19 (70)	1/19 (5.3)	
Dressings	4 (21)	0/4 (0)	
NPWT	15 (79)	1/15 (6.7)	
Surgical management (SM)	8 (30)	2/8 (25.0)	
Local flap	7 (88)	2/7 (28.6)	
Free flap	1 (12)	0/1 (0)	
Hardware-preservation outcome			
Success	24 (89)		
Failure requiring revision	3 (11)		
Final functional recovery			
Complete	18 (67)		
Partial	8 (30)		
None	1 (3)		
Follow-up			
> 2 years	11 (41)		

Exploratory statistical analysis did not identify any variable significantly associated with failure at the p < 0.05 threshold. However, time to treatment greater than one week showed the most pronounced trend, with a failure rate of 25.0% (2/8) compared to 5.3% (1/19) for patients treated within one week (Fisher’s exact test, p = 0.201; odds ratio 6.0). All three failures occurred at the malleolar site (3/16, 18.8%) versus no failures at non-malleolar sites (0/11, 0%), though this difference did not reach statistical significance (Fisher’s exact test, p = 0.248). Exposure size greater than 1 cm was not significantly associated with failure (3/23 vs 0/4; Fisher’s exact test, p = 1.000), likely reflecting insufficient statistical power due to the small number of patients with exposures below 1 cm. No association was found between failure and smoking (*p* = 0.569), diabetes (*p* = 0.532), peripheral vascular disease (*p* = 0.596), sex (*p* = 1.000), or age (median 66 vs 74.5 years in failures vs successes; Mann–Whitney U, *p* = 0.313). The number of cumulative risk factors did not show a dose–response relationship with failure (Table 3, 4 and 5).

## Discussion

### General considerations

The orthoplastic approach entails coordinated efforts between orthopaedic and plastic surgeons to address acute bone trauma when soft tissue status is compromised or unclear [9–12]. However, assessing soft tissue condition can be challenging, operator-dependent, and not always feasible in the acute care setting in the presence of plastic surgeons. Consequently, plastic surgeons often intervene at a later stage. In this context, it is crucial to ensure optimal conditions for their involvement by minimizing the risk of progression from contamination to established infection, particularly in cases where dehiscence is not of infectious aetiology.

The available literature regarding optimal treatment for orthopaedic hardware exposure lacks a uniform level of evidence due to the predominantly retrospective and descriptive nature of existing studies. Consequently, the current evidence landscape is heterogeneous, and higher-quality research is needed to establish more definitive recommendations [1, 3, 13].

**Fig. 3:**
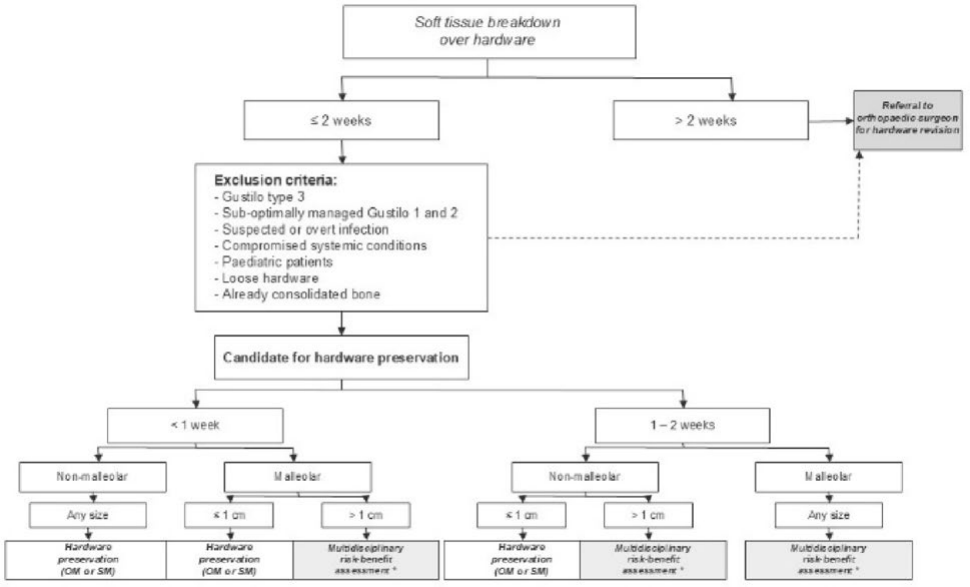
Proposed decision-making pathway for hardware preservation in cases of soft tissue breakdown over exposed osteosynthesis. This pathway is based on exploratory observations from the present series and requires prospective validation

In the absence of specific guidelines, clinical decision-making at our institution relies on available data combined with principles of good clinical practice. The pathway supporting treatment choice—whether hardware removal or hardware preservation—is based on a multidisciplinary evaluation of patient-, trauma-, and procedure-related variables. The present study was designed as a preliminary investigation to describe the outcomes of this institutional approach and to explore whether specific variables may be associated with treatment success or failure. Three variables emerged as potentially relevant from the descriptive and exploratory analysis: exposure size, time to treatment, and exposure site (Fig. 3). However, given the small sample size (n = 27, 3 failures), these associations should be interpreted as hypothesis-generating observations rather than validated predictors.

### Risk factors assessment

The relatively high median age of the cohort (74 years) deserves comment. Typically, patients experiencing limb trauma tend to be younger. The age distribution observed in this series likely reflects a selection effect: older patients with soft tissue fragility and comorbidities may be more prone to wound dehiscence over hardware, and the preference for avoiding a second major surgical procedure may have further favoured a hardware-preservation strategy in this population. It should also be noted that age was not associated with failure in this series (median 66 vs 74.5 years in failures vs successes; Mann–Whitney U, *p* = 0.313), although the sample is too small to draw conclusions from this observation.

The cutoffs used to categorize exposure timing and size were defined a priori by the authors for exploratory purposes and should not be interpreted as data-driven thresholds. For timing, the literature primarily addresses a two-week boundary for definitive treatment [3]. Since a delay longer than 2 weeks already constituted an exclusion criterion for hardware preservation in this series, the authors elected to explore differences within this window (i.e. less than 1 week versus between 1 and 2 weeks).

Similarly, the authors categorized the exposure size into three groups: less than 1 cm, between 1 and 3 cm, and greater than 3 cm. There are currently no clear indications in the literature regarding the role of exposure dimensions in determining outcomes, and, to the authors’ knowledge, no previous study has proposed a categorization of exposure size for the purpose of evaluating its association with treatment success or failure. These cutoffs are therefore exploratory and require external validation in larger, more homogeneous cohorts.

A notable and somewhat counterintuitive finding was the absence of any association between traditional vascular risk factors—smoking, diabetes, and peripheral vascular disease—and treatment failure. None of the 8 diabetic patients failed, and the cumulative number of risk factors showed no dose–response relationship with failure. This likely reflects a selection bias inherent to the study design: patients with more severe comorbidity profiles and larger or more complex exposures were presumably channelled towards hardware removal at the multidisciplinary evaluation stage, leaving a relatively “favourable” subset within the hardware-preservation cohort. This confounding by indication limits the interpretability of risk factor data in the present series and underscores the need for prospective studies with systematic documentation of the entire population of patients presenting with hardware exposure, including those who undergo revision.

### Failure analysis

The exploratory statistical analysis identified time to treatment as the variable showing the most pronounced, though non-significant, trend towards association with failure. Patients referred after one week had a failure rate of 25.0% (2/8) compared to 5.3% (1/19) for those treated within one week, with an odds ratio of 6.0 (Fisher’s exact test, *p* = 0.201). This finding is biologically plausible: the delay in initiating treatment exposes patients to a higher risk of biofilm maturation and subclinical deep infection, potentially leading to false negatives on superficial microbiological swabs and ultimately to failure of hardware preservation due to the presence of an underlying undiagnosed infectious process.

All three failures occurred at the malleolar site (3/16 malleolar exposures, 18.8%) versus no failures among 11 non-malleolar exposures (Fisher’s exact test, *p* = 0.248). This concentration, while not statistically significant, is consistent with the well-documented challenges of healing in the distal lower extremity [14], where the soft tissue envelope is thin, vascularity is precarious, and mechanical stresses are considerable—even in the absence of other known risk factors [15–17]. All three failures also involved exposures larger than 1 cm. The three-category breakdown showed a gradient in failure rates (0% for exposures below 1 cm, 10% for 1–3 cm, 33.3% for those exceeding 3 cm), suggesting that larger exposures may increase the surface area available for bacterial colonization and impair the capacity of local tissues to achieve secondary coverage. However, the small number of patients in the extreme categories (4 below 1 cm, 3 above 3 cm) prevents any meaningful statistical evaluation of this trend (Fisher’s exact test for above vs below 1 cm, *p* = 1.000) [18–20].

When these three variables are considered together, a pattern emerges from the data. Among patients treated within one week at non-malleolar sites, regardless of exposure size, no failures were recorded. Conversely, the combination of malleolar location, exposure exceeding 1 cm, and referral after one week characterized two of the three failures. The remaining failure occurred at a malleolar site within one week but in the presence of peripheral vasculopathy, suggesting that site-specific vascular compromise may represent an additional contributing factor. These observations are consistent with the hypothesis that the window between 7 and 14 days after exposure detection represents the most critical interval for clinical decision-making, where the interplay of anatomical location, exposure dimensions, and local tissue conditions should most carefully inform the choice between hardware preservation and revision. However, these patterns derive from a descriptive analysis of only 3 events in 27 patients and must be considered tentative until tested in adequately powered studies.

### Final considerations

**Fig. 4:**
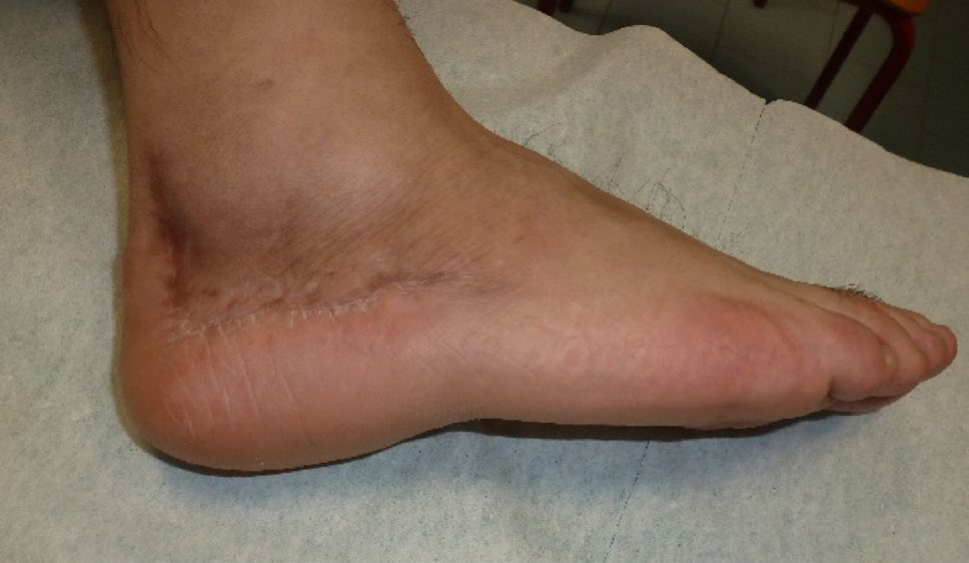
Healed lateral malleolus wound following hardware preservation with outpatient management (OM)

**Fig. 5:**
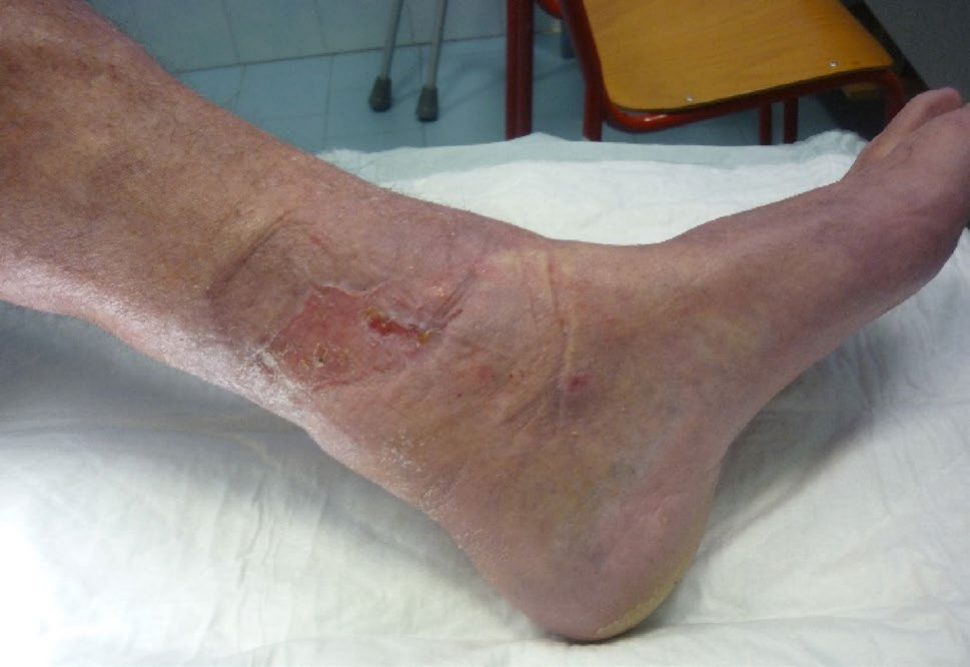
Healed medial distal leg wound following hardware preservation with surgical management (SM), consisting of negative pressure wound therapy (NPWT) followed by split-thickness skin graft (STSG). Pre-treatment photographs were not available for this case

Despite the acknowledged limitations—including its retrospective design, single-centre setting, cohort heterogeneity in terms of anatomical sites and treatment modalities, selection bias inherent to the multidisciplinary triage process, and the overlap with the COVID-19 pandemic—this study describes a series in which positive outcomes were achieved with a hardware-preservation strategy in carefully selected patients. In 24 of 27 cases, it was possible to avoid renewing bone synthesis, minimizing additional bone tissue manipulation and associated costs, with potential benefits for both patients and the healthcare system (Figs. 4 and 5).

An important limitation is the incomplete long-term follow-up: only 11 out of 27 patients have reached the 2-year mark. This is particularly relevant because chronic subclinical osteomyelitis can emerge months to years after the initial episode [21]. While none of the patients in this series have shown signs of chronic osteomyelitis to date, the absence of systematic long-term surveillance in the remaining 16 patients means that late infectious complications cannot be definitively excluded.

Furthermore, for patients who undergo ORIF with plates and screws, successful outcomes entail bone healing without complications, rapid functional recovery, and the absence of residual pain [6, 22]. In this series, hardware-preservation success was achieved in 24 of 27 cases (89%). Final functional recovery, including patients who subsequently required hardware revision, was observed in 26 of 27 cases (97%). Should the need arise, removal of the synthesis material can be performed once bone healing is achieved, without delaying limb function recovery.

The cohort heterogeneity—encompassing five different anatomical sites, two treatment modalities (OM and SM) with further subdivisions, and varying exposure characteristics—represents both a strength and a limitation of this work. It reflects real-world clinical practice, where decision-making must accommodate diverse presentations; however, it also prevents the identification of site-specific or treatment-specific predictive factors. Future studies should ideally focus on a single anatomical district, most logically the malleolar region given its high prevalence in this series and its association with all observed failures.

These preliminary data suggest several directions for further research: confirmation or refutation of the observed trends through larger multicentre series with standardized inclusion criteria and long-term follow-up; formal multivariable analysis of the emerging risk factors once adequate sample sizes are achieved; cost-effectiveness analysis comparing hardware preservation with hardware removal and revision; and prospective validation of the proposed cutoffs for exposure timing and dimensions. The risk stratification based on exposure size, timing, and anatomical location described in this series could serve as the starting point for the design of such studies, rather than as a definitive treatment algorithm in its current form.

## Conclusions

Soft tissue breakdown over orthopaedic hardware presents a persistent and challenging clinical problem, often necessitating hardware removal and revision of bone fixation, which prolongs healing times and increases costs. In this preliminary retrospective series, selected patients with mechanically stable osteosynthesis, no overt clinical signs of infection, negative superficial wound swabs, and referral within two weeks of exposure detection were managed using a hardware-preservation strategy focused on soft tissue coverage under systemic antibiotic therapy. Stable coverage without hardware removal was achieved in 24 of 27 cases.

Exploratory analysis identified three variables potentially associated with treatment outcome: time from exposure detection to treatment, exposure dimensions, and anatomical location. Failures clustered in malleolar exposures larger than 1 cm, particularly when referral occurred after one week; however, none of the explored associations reached statistical significance. Particular attention should be exercised when considering hardware preservation for plate exposure in the malleolar region, especially if accompanied by size exceeding 1 cm or time to treatment greater than 1 week.

Given the small sample size, the retrospective single-centre design, cohort heterogeneity, and incomplete long-term follow-up, these findings should be regarded as hypothesis-generating. Larger prospective multicentre studies, ideally focused on anatomically homogeneous cohorts, are required to validate timing- and size-based thresholds and to determine whether a reproducible decision-making framework can be developed for hardware-preserving management of exposed stable osteosynthesis.

## Data Availability

The datasets generated and analysed during the current study are not publicly available due to patient privacy and confidentiality restrictions, but anonymised data may be made available from the corresponding author upon reasonable request and subject to institutional approval.

## Abbreviations

COVID-19: Coronavirus disease 2019
NPWT: Negative pressure wound therapy
OM: Outpatient management
ORIF: Open reduction internal fixation
SM: Surgical management
STSG: Split-thickness skin graft
